# Development of an LC-MS method for determination of nitrogen-containing heterocycles using mixed-mode liquid chromatography

**DOI:** 10.1007/s00216-020-02665-x

**Published:** 2020-05-26

**Authors:** Mohammad Sajjad Abdighahroudi, Holger V. Lutze, Torsten C. Schmidt

**Affiliations:** 1 Faculty of Chemistry, Instrumental Analytical Chemistry, University of Duisburg-Essen, Universitätsstraße 5, 45141 Essen, Germany; 2 IWAR, Technical University Darmstadt, Franziska-Braun Str. 7, 64287 Darmstadt, Germany; 3 IWW Water Centre, Moritzstraße 26, 45476 Mülheim an der Ruhr, Germany; 4 Centre for Water and Environmental Research (ZWU), Universitätsstraße 5, 45141 Essen, Germany

**Keywords:** Nitrogen-containing heterocycles, Liquid chromatography, Reversed-phase, Cation exchange, Mixed-mode chromatography

## Abstract

*N*-containing heterocycles (NCHs) are largely used as precursors for pharmaceuticals and can enter the environment. Some NCHs have been shown to be toxic, persistent, and very mobile in the environment. Thus, they have received increasing attention in the past years. However, the analysis of these polar compounds in environmental samples is still a challenge for liquid chromatography. This paper investigates the use of mixed-mode liquid chromatography (MMLC), which has reversed-phase and ion exchange characteristics for measurements of NCHs in water. NCHs with low p*K*_a_ (i.e., < 2.5) display mainly reversed-phase interactions (neutral species) with the stationary phase and those with higher p*K*_a_ (i.e., > 5) interact by a mixture of reversed-phase/ion exchange/HILIC mechanism. It was also shown that the presented method performs well in the quantification of the majority of the selected NCHs in surface water with MDLs between 3 and 6 μg/L, a low matrix effect and recoveries in the range of 77–96% except for pyridazine exhibiting 32% were achieved. The method was successfully employed to follow the degradation of NCHs in ozonation.

**Electronic supplementary material:**

The online version of this article (10.1007/s00216-020-02665-x) contains supplementary material, which is available to authorized users.

## Introduction

*N*-containing heterocycles (NCH) are a group of compounds with wide occurrence in the environment and broad application in chemistry [1–3]. They have been detected at industrial and agricultural sites [4, 5], groundwater [6–10], and surface water [11, 12]. They were also found in coal liquids, shale oil, surface waters such as lakes, and marine sediments [13]. The features of chemicals such as pharmaceuticals, industrial solvents, ionic liquids, and pesticides are based on NCH moieties [14–16]. Important moieties of, e.g., pharmaceuticals, are piperidine, pyridine, and imidazole [17]. Aromatic *N*-heterocycles such as pyrazole, pyridine, and pyrazine are also by-products of chemical processes such as acrylonitrile manufacturing [18].

Many NCHs are mutagenic, carcinogenic, and thus, hazards to the environment and human health [19], e.g., pyridine is toxic for several bacterial species and a strong odor compound [20]. It can cause weakness, lung and liver damage, and gastrointestinal inflammation in humans by acute exposure [15]. NCHs such as pyridine and imidazole are categorized as water hazard category 2 (significantly hazardous to the aquatic environment), and pyrazole is categorized as water hazard category 3 (highly hazardous to the aquatic environment) by German Environment Agency (Umweltbundesamt – UBA) (Table 1) [22]. Hence, UBA has set a provisionary water standard of 3 μg/L for pyrazole [24].

**Table 1:** Physical and chemical properties of selected N-heterocycles

Compound	Mw	[M+H]^+^(m/z)	Structure	p*K*_a_^a^ [21]	log P [21]	log (D)^b^	Water hazard category [22]
Imidazole	68.07	69.07		6.99	-0.08	-0.38	2
Pyrazole	68.07	69.07		2.49	0.33	0.33	3
Pyridine	79.10	80.10		5.23	0.65	0.64	2
Pyridazine	80.09	81.09		2.24	-0.72	-0.72	Not Available
Piperidine	85.15	86.15		11.123	0.85	-3.27	1

^a^Conjugated acid

^b^At pH 7 calculated based on [23]

Due to their basic properties (Table 1), NCHs are readily water soluble as cations at typical pH values of surface and groundwaters and thus mobile in the aquatic environment. NCHs are not directly photo-oxidized due to low absorption maxima [8], and some of them, such as pyrazole, are poorly biodegradable [25]).

To abate NCHs, different methods can be utilized [12, 15, 19]. Ozonation is one of the methods that is extensively used to eliminate NCHs, whether in wastewater [12] or process studies [26, 27]. Monitoring these methods and processes requires specific and sensitive measurement of NCHs.

The high affinity of NCHs towards the aqueous medium results in a challenge for classical reversed-phase liquid chromatography (RPLC) separation techniques [28]. For polar and small compounds such as imidazole, piperidine, and pyridine, there is hardly any retention on C18 columns [14, 29, 30]. This causes co-elution with non-retarded matrix components present in the sample, such as salts, and thus interferences in the detection of these NCHs (e.g., spectral interference (UV-vis detection) or ionization (MS-detection)) [31]. Moreover, solid-phase extraction (SPE) hardly enriches polar compounds such as NCHs [32–34]. Using ion chromatography (IC) [35] or ion pair chromatography [36] retention of NCHs was accomplished. However, the use of nonvolatile buffers, necessity for strong ion-pairing agents, and high water percentage render hyphenation with MS challenging, which is the detection method of choice.

Recently the use of mixed-mode liquid chromatography (MMLC) was suggested for the separation of polar and charged compounds as an alternative to hydrophilic interaction liquid chromatography (HILIC) [14, 37]. MMLC provides different modes of interactions with the analytes, such as hydrophobic interaction and ion exchange [38]. This can be used for separation of analytes with very different properties. For example, MMLC columns with anion or cation exchangers embedded in their hydrophobic alkyl chains [37] were used for the simultaneous measurement of cationic, zwitterionic, and neutral compounds previously [39]. Moreover, obstacles such as long equilibration time and use of organic solvent in the sample do not exist in MMLC compared to HILIC [33, 34]. However, up to now, MMLC was not used for the determination of NCHs.

The present work aims to develop an MMLC-MS method for the analysis of NCHs in water samples. It furthermore characterizes the separation mechanisms and employs the method in measurement of selected NCHs in the river water matrix. Applicability of the method in process monitoring will be tested for ozonation of the same water matrix.

## Material and methods

### Chemicals

All chemicals were commercially available and used as received (purity is presented in parenthesis): piperidine (ReagentPlus®, 99%) Sigma-Aldrich, pyridine (anhydrous, 99.8%) Sigma-Aldrich, imidazole (ACS reagent, ≥ 99%) Sigma-Aldrich, pyrazole (98%) Sigma-Aldrich, pyridazine (98 + %) Alfa Aesar. Water for chromatography (LC-MS Grade) LiChrosolv Merck Millipore, acetonitrile (ACN) HiPerSolv CHROMANORM® for LC-MS (≥ 99.9%,) VWR Chemicals, formic acid Suprapur (98–100%) Merck Millipore, nitrogen gas (> 99%) and oxygen gas (99.998%) from Air Liquid, Oberhausen.

### Instrumentation

The following instruments were used: pH meter from Metrohm 827pH, TOC analyzer from Shimadzu TOC-L with ASI-L autosampler, and TOC Control-L Software. HPLC from Agilent Technologies (1100 Series) with Autosampler coupled with a Quadrupole LC/MS 6120 Mass spectrometer. LC-MS online software was used for data acquisition and processing. ESI was operated as follows: API-ES-positive mode, drying gas (nitrogen) temperature 250 °C, drying gas flow rate 11.0 L/min, nebulizer pressure 35 psi, capillary voltage 3.2 kV. The temperature of the column oven was kept constant at 30 °C in all measurements, and the flow rate was 300 μL/min with 5 μL injection volume. A Primesep 200 column (2.1 × 150 mm 5-μm particle size and 100-A pore size) and guard column (2.1 × 5 mm) from SIELC technology were used for separation. Single ion monitoring was used to measure real water samples. For further details on MS parameters, see Electronic Supplementary Material (ESM) Table S4.

Ozone was generated by continuous purging of ozone enriched O_2_ stream into ultrapure water, using a Philaqua 802 x ozone generator from BMT Messtechnik Berlin. The concentration of ozone was determined by the determination of UV-absorption (ε_ozone_ at 258 nm = 2950 M^−1^ cm^−1^ [40]) using a Shimadzu UV-1800 spectrometer. The used absorption coefficient is different from the more recently published one of 3200 M^−1^ cm^−1^ [41]; however, it includes losses of ozone happening during the transfer of the ozone stock solution to the UV-vis spectrometer.

### Chromatography

For optimization of the separation using MMLC, one has to determine the main separation modes. Based on reversed-phase (RP) interaction, an increase of the organic phase volume fraction (here ACN), and thus a decrease of aqueous volume fraction, results in a decrease of the retention factor (Eq. 1) [42].

1 $$\log \mathrm{k}^{\prime }=-\mathrm{S}\times {\updelta}_{\mathrm{A}\mathrm{CN}}+\log \mathrm{k}^{\prime}_{\mathrm{A}}$$

where k′ is the observed retention factor, δ_ACN_ is the volume fraction of ACN, *S* is the slope the linear line, k′_A_ is the retention factor when the volume fraction of ACN is zero, while for ion exchange (IE) chromatography, the retention factor is based on Eq. 2 [42].

2 $$\log \mathrm{k}^{\prime }=-\mathrm{Z}\times \log\ {C}_s+\log \mathrm{k}^{\prime}_z$$

where *C*_s_ is the concentration of counter ion (hydrogen ion), *Z* is the slope of the linear line, and k′_z_ is a constant related to ion exchange properties of eluent and stationary phase [43].

To investigate the role of both IE and RP interactions for retention of different NCHs, a 1000 μg/L sample was analyzed using isocratic conditions with different δ_ACN_. In these experiments, the water was acidified with formic acid (FA), but ACN was not acidified. By mixing not acidified ACN with acidified water up to 60%, the final pH of the eluent will increase linearly according to Eq. 3 [44].

3 $${}_{\mathrm{w}}^{\mathrm{s}}\mathrm{pH}={}_{\mathrm{w}}^{\mathrm{w}}\mathrm{pH}+{m}_{\mathrm{pH}}\times {\updelta}_{\mathrm{ACN}}$$

where ${}_{\mathrm{w}}^{\mathrm{s}}\mathrm{pH}$ is the pH of the mixture, ${}_{\mathrm{w}}^\mathrm{w}\mathrm{pH}$ is the initial pH of the water before mixing with ACN, and *m*_pH_ is the proportionality coefficient for the pH variation. *m*_pH_ also depends on the concentration of acid and initial pH, which is discussed by Subirats et al. [44]. As a result of increasing pH, (decrease of formic acid concentration in the final mixture of eluent by increasing δ_ACN_ and decrease of hydrogen ion as the counter ion), the IE interactions should become weaker by the increase of δ_ACN_ in these experiments, i.e., the retention behavior will be contrary to RP interaction. The investigation of the separation performance was based on parameters shown in ESM Table S1.

### Method validation

To investigate the linearity of the method, three series of 10 samples with concentrations 1, 2, 5, 10, 20, 50, 100, 200, 500, 1000 μg/L were chosen based on DIN 38402–51 [45] and analyzed. Mandel’s test was calculated based on [46]. In brief, an *F* test was performed to compare linear and second-order polynomial calibration according to the following equation:

4 $$F=\frac{\left(n-2\right)\times {S_{y/x, lin}}^2-\left(n-3\right)\times {S_{y/x, non}}^2}{{S_{y/x, non}}^2}$$

where *n* is the number of calibration points; S_y/x,lin_ and S_y/x,non_ are the standard error of the linear and nonlinear regression, respectively.

The method detection limit was quantified according to EPA [47] by analyzing nine different samples containing a mixture of all compounds, each with a concentration of 25 μg/L. Measurements were performed in 3 different batches in 3 different days and method detection limit (MDL) was calculated according to Eq. 5:

5 $$\mathrm{MDL}={t}_{\left(n-1,\kern0.75em 1-\alpha =0.99\right)}\times {S}_S$$

where *t*_(*n* − 1, 0.99)_ is Student’s *t* value for a single-tailed 99th percentile (2.896 for *n* = 9), and *S*_s_ is the standard deviation of measured samples.

The performance of the method was investigated in river water. The river water was filtered by 0.45-μm cellulose acetate membranes and stored in the refrigerator at ≈ 5 °C until further use. Loss of the polar NCHs, e.g., due to sorption in this step, is negligible [33]. The sample had a TOC concentration of 3.53 mg/L and a pH of 7.87. Portions of 10 mL surface water samples were spiked with different volumes of the stock solution of the NCHs (mixture, dissolved in water, concentration: 10 mg/L) to prepare six different concentration levels. These samples were measured in three non-consecutive days, each in triplicate using freshly made eluent by the same operator. Recoveries were calculated as the average of all recoveries within the whole method validation studies. By utilizing analysis of variance (ANOVA), the variation of different performance parameters within a day (repeatability) and between days (intermediate precision) were calculated according to the Eurachem Guide [48].

The matrix effect was assessed by comparing the slope of spiked river water samples with ultrapure water samples according to Eq. 6.

6 $$\mathrm{Matrix}\ \mathrm{effect}=\left(\frac{\mathrm{Slope}\ \mathrm{in}\ \mathrm{spike}\ \mathrm{samples}}{\mathrm{Slope}\ \mathrm{in}\ \mathrm{ultrapure}\ \mathrm{water}}-1\right)\times 100$$

## Results and discussion

### Method development

Void time was determined by injecting NaBr and measurement of bromide at different ACN volume fractions (δ_ACN_) in negative ionization mode. The experiment was performed at the same chromatographic conditions used for determination of chromatographic behavior (section 2.2). As expected, no trend in void time was observed regarding δ_ACN_, ranging between 1.19 and 1.20 min.

**Fig. 1:**
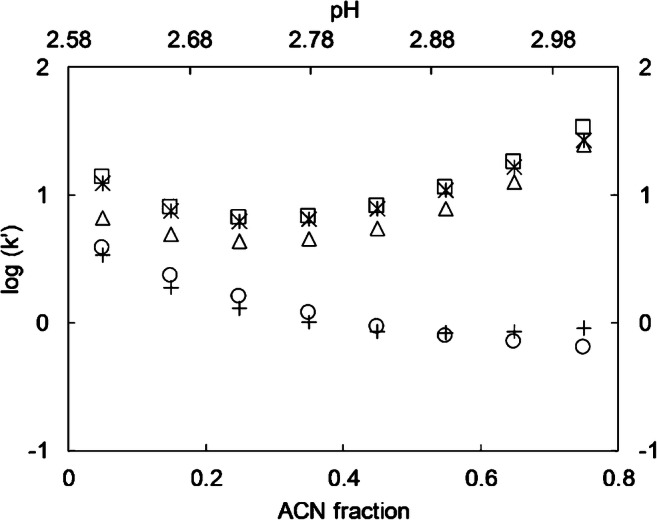
Logarithm of retention factor (log(k′)) in isocratic elution of NCHs at different ACN volume fractions and the pH of the final eluent mixture. Imidazole (triangles), pyrazole (circles), pyridine (stars), pyridazine (crosses), and piperidine (squares). Eluent A: water +0.1% FA, Eluent B: ACN. Flowrate 300 μL/min, 5 μL injection volume. Separation column: Primesep 200

Using the void time and retention times, retention factors were calculated at different δ_ACN_ (Fig. 1). It was observed that an increase of δ_ACN_ leads to a decrease in retention time and log(k′) for pyridazine and pyrazole. Therefore, these two compounds mainly interact via RP mode. It is also worth mentioning that at lower δ_ACN_ pyrazole elutes later than pyridazine, while at higher fractions, the order of elution changes. On the other hand, the stronger bases imidazole, pyridine, and piperidine (c.f. p*K*a values, Table 1) display a first decrease of the retention factor until δ_ACN_ reaches 0.25. At δ_ACN_ > 0.25 retention factor increases. This indicates that at δ_ACN_ ≤ 0.25, these three compounds interact via RP interactions and at higher δ_ACN_ ≥ 0.25 via ion exchange interactions. This can be explained as follows. Pyridazine and pyrazole are mainly present as neutral species during the whole range of δ_ACN_, and thus, hydrophobic interactions may be most important. In the case of imidazole, pyridine, and piperidine, cationic species prevail in the whole range of applied δ_ACN_ and can thus be separated by ion exchange interactions. The minimum of log(k´) δ_ACN_ at 0.25 indicates that imidazole, pyridine, and piperidine also interact by the RP mode of the MM-column, which are, however, suppressed with increasing ACN fractions and cannot be observed at δ_ACN_ > 0.25. The exemplary chromatogram in ESM Fig. S1 illustrates how the retention time of imidazole and pyrazole increases and decreases by the increase of δ_ACN_, respectively.

Efficiency and asymmetry are almost constant for imidazole, pyridine, and piperidine for all δ_ACN_, except for 0.05 in which pyridine and piperidine show somewhat lower efficiency (ESM Figs. S2 and S3). On the other hand, for pyridazine and pyrazole, efficiency decreases with increasing δ_ACN_. Higher peak widths are also observed for imidazole, pyridine, and piperidine congruent with higher retention time (ESM Fig. S4). Moreover, by comparing the selectivity (ESM Fig. S5) with the resolution (ESM Fig. S6) one can observe that they follow the same trend. It appears that the role of selectivity in the calculation of resolution (formula presented in ESM Table S1) is greater than the retention factor (Fig. 1) and efficiency (ESM Fig. S2).

Figure 1 also represents the relation between log(k) and pH. According to Eq. 3 [44] the increase in the pH of final the eluent mixture is linear up to δ_ACN_ = 0.6. Therefore, the retention of the analytes with IE interaction should also increase, as H^+^ competes with the cationic analytes for ion exchange moieties (Eq. 2). Moreover, pyrazole and pyridazine are mostly present as neutral species in the pH between 2.6 to 2.9 and thus interact with RP. On the other hand, imidazole, pyridine, and piperidine are present as cations in the pH range of the eluent mixture and thus, the role of IE interaction is relevant for them. The U-shaped correlation of pH vs. log(k) for piperidine, pyridine, and imidazole indicates that two interaction modes work at the same time (i.e., RP and IE interactions) while in the range of linear increase at higher δ_ACN_, IE interactions prevail. This type of U-shaped retention behavior is known to happen in HILIC phases [49] and was also previously observed in MMLC using other mixed-mode stationary phases [14, 50]. Montes et al. [38] described this as showing RP retention at low δ_ACN_ and HILIC-like retention at high δ_ACN_ for MMLC.

**Fig. 2:**
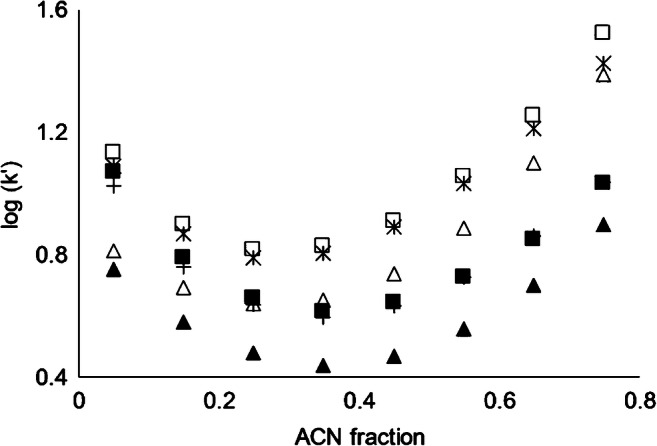
Comparison between logarithm of retention factor (log(k′)) using two different eluent compositions. (1) Eluent A: water +0.1% FA, Eluent B: ACN, imidazole (triangles) pyridine (stars), and piperidine (squares). (2) Eluent A: water + 0.1% FA, Eluent B: ACN + 0.2% FA imidazole (filled triangles), pyridine (crosses), and piperidine (filled squares). Flowrate 300 μL/min, 5 μL injection volume. Separation column: Primesep 200

In another set of experiments, both components of the eluent, i.e., water and ACN, were acidified with 0.1% and 0.2% FA, respectively in order to investigate the suppression of IE at higher δ_ACN_. Compared to the eluent with non-acidified ACN, the increase of retention factor at δ_ACN_ > 0.45 is less pronounced (Fig. 2). This can be explained by a constant elution strength for the IE mechanisms which corroborates that IE is indeed an important separation mechanism in the case of imidazole, pyridine, and piperidine. Substantial decrease of retention time by the increase of FA concentration for amines with p*K*_a_ values of the corresponding acids above 8 was also previously observed by other researchers [39]. On the other hand, the hydrophilic interactions that are relevant in HILIC seem to be not completely suppressed, leading to the observed increase of the retention at δ_ACN_ > 0.45. Contrary to RP and IE interactions (Eqs. 1 and 2), the relation between log(k′) and δ_ACN_ in HILIC interactions is not first order. HILIC is modeled as having RP interaction in low δ_ACN_ and normal phase (NP) interaction at high δ_ACN_ according to [49].

7 $$\log \mathrm{k}^{\prime }={m}_1\times \left(1-{\updelta}_{\mathrm{ACN}}\right)-{m}_2\times \log\ \left(1-{\updelta}_{\mathrm{ACN}}\right)+\mathrm{constant}$$

where *m*_1_ and *m*_2_ are the empirical constants related to RP and NP interactions, respectively. Equation 4 can well describe the chromatographic behavior of all the compounds under investigation. Calculated parameters are presented in Table S2 and Fig. S7 (see ESM) represents the modeled data. All in all, it can be confirmed that NCHs that have basic properties follow a separation based on three interactions (RP/IE/HILIC). These multiple interactions provide many options to improve a separation from compounds which have fewer interactions with the stationary phase.

Based on the above data, a method was developed for the detection of these NCHs in a surface water matrix to achieve proper retention and adequate separation from other constituents of the sample to avoid interferences during ESI [51]. The chromatographic method optimization was performed to achieve the best peak shape in a short runtime (k′, 2 to 5). As can be seen from Fig. 2, k′ of 5 (log(k′) ≈ 0.7) can be achieved for stronger bases only with the second set of eluents and at the middle δ_ACN_. Using different compositions of the mobile phase and gradient elution, the final method was developed according to ESM Table S3. MS measurements were done in two modes: in single ion monitoring (SIM) to improve the sensitivity and in scan mode (ESM Table S4).

Using the abovementioned conditions, a separation with parameters shown in Table 2 was achieved. Under these conditions, the efficiency of pyridine and piperidine separation largely increased, while the other compounds did not change much compared to the previous measurements. The method performance is very good regarding peak width (13 to 18 s) and asymmetry factors of maximum 1.65, particularly taking into account that the best peak shapes were obtained for the compounds with no retention in RP (i.e., imidazole, pyridine, piperidine).

**Table 2:** Method performance parameters for measurement of NCHs. Flowrate 300 μL/min, 5 μL injection volume. Separation column: Primesep 200, Gradient according to ESM Table S3. MS conditions according to ESM Table S4

Compound	Retention (*t*_R_) [min]	Peak width (*W*_50_) [min]	Peak asymmetry (*A*_s_)	Retention factor (k′)	Selectivity (*α*)	Efficiency (N) [plates/column]	Resolution (*R*_s_)
Imidazole	8.93	0.29	1.03	3.65	1.74	4399	5.55
Pyrazole	5.94	0.26	1.65	2.09	1.18	2960	1.43
Pyridine	12.48	0.22	1.27	5.50	1.51	17424	9.40
Pyridazine	5.31	0.24	1.32	1.77	–	2410	–
Piperidine	12.92	0.23	1.16	5.73	1.04	17062	1.12

### Method validation

Table 3 shows Mandel’s test results for linearity and method detection limit (MDL). For all the compounds, calibrating by consideration of the highest investigated concentration (1000 μg/L) leads to a lack of linearity according to Mandel’s test. Therefore, 500 μg/L is the highest linear point in the logarithmic range.

**Table 3:** Method performance parameters for measurement of NCHs. Flowrate 300 μL/min, 5 μL injection volume. Separation column: Primesep 200, Gradient according to ESM Table S3. MS condition according to ESM Table S4

Compound	Slope (sensitivity) (peak area × L/μg)	Intercept (peak area)	*R*^2^	Method standard deviation (μg/L)	Linear range (μg/L)	MDL (μg/L)
Imidazole	(69.8 ± 0.2) × 10^3^	(− 1 ± 0.4) × 10^5^	0.9999	1.55	1–500	6.11
Pyrazole	(81.7 ± 0.4) × 10^3^	(− 2.2 ± 0.8) × 10^5^	0.9998	2.44	1–500	3.62
Pyridine	(186.8 ± 0.8) × 10^3^	(− 0.6 ± 0.1) × 10^5^	0.9999	1.88	1–500	3.3
Pyridazine	(59.4 ± 0.8) × 10^3^	(− 4.7 ± 1.6) × 10^5^	0.9991	6	5–500	4.93
Piperidine	(174.1 ± 0.7) × 10^3^	(− 4.7 ± 1.3) × 10^5^	0.9999	1.93	1–500	2.74

All the measured MDLs are between 3 and 6 μg/L, according to Table 3. It should be noted that no peaks were observed in blank samples; hence, no correction of MDL was required. For pyridine the detection limit of a previously developed method was reported to be 4.6 μg/L achieved by extraction of 1 L water into 1 mL methylene chloride followed by GC-MS [52]. Using electrical field–stimulated liquid-phase microextraction followed by HPLC-UV [53], 0.01 μg/L MDL was also achieved for pyridine. Pyrazole has been measured using vacuum-assisted headspace solid-phase microextraction followed by GC-MS (LOQ = 0.04 μg/L) [54], or by injecting sample in volumes as high as 90 μL in LC followed by MS in multiple reaction monitoring (0.05 μg/L) [55]. Utilizing conductivity detector and RP columns provided detection limits for ionic liquids of piperidinium, pyridinium, and imidazolium in the milligram per liter range [56, 57]. It can be stated that the MMLC method is similarly sensitive, considering that it could be improved by a factor of 1000 using an appropriate extraction method. The method standard deviation (residual standard deviation divided by slope) is similar for most of the investigated compounds. Pyridazine, however, showed an approximately three times higher method standard deviation than the average.

Table 4 shows method performance in river samples. The recoveries for imidazole, pyridine, and piperidine are more than 90%, while pyridazine showed a recovery of ca. 30%. In a similar manner, a relatively small matrix effect is observed in the measurement of NCHs except for pyridazine (ESM Fig. S8). One reason for the small matrix effect can be the low flow rate in this method, which can reduce matrix effects in ESI [51]. This can be further improved by post-column splitting [58] or using smaller dimensions of the column with proportionally lower flow rate. Moreover, using other ionization sources such as APCI [59] and APPI [60] might also help to reduce the matrix effect as they are less prone to interferences. The high matrix effect for pyridazine affected the overall recovery of the compound. It can be stated that the method is not performing well for the detection of pyridazine in the tested river water matrix.

**Table 4:** Recovery, repeatability, and intermediate precision for measurement NCHs in spiked surface water. Flowrate 300 μL/min, 5 μL injection volume. Separation column: Primesep 200, Gradient according to ESM Table S3. MS condition according to ESM Table S4

Compound	Average recovery (%)	Repeatability (%RSD)	Intermediate precision (%RSD)
Imidazole	92	1.1	5.17
Pyrazole	77	3.12	6.82
Pyridine	96	0.92	4.09
Pyridazine	32	7.21	26.66
Piperidine	91	0.74	4.69

Moreover, higher intermediate precision in Table 4 can be attributed to a higher deviation from actual values in different days due to further variations of solvents, (room) temperatures, performance of the instrument (e.g., condition of seals, capillaries), etc. in comparison to triplicate injection within a day. While the intermediate precision for NCHs except pyridazine was ca. 5.2%, repeatability had a clear better performance for compounds with small or no tailing, i.e., imidazole, pyridine, and piperidine.

Pyridazine also showed worse performance in terms of retention time, peak width, and peak asymmetry with over 20% RSD (Table 5). Moreover, a difference between repeatability and intermediate precision of retention times was observed for all compounds due to the decrease in retention time. However, both within-a-day and between-days variation are very small in absolute terms, except for pyridazine. The highest intermediate precision (0.4% for imidazole) originates from the highest decrease of 4 s in retention time in three non-consecutive days. The peak width and asymmetry had no significant difference in intraday and interday measurements for NCHs other than pyridazine.

**Table 5:** Repeatability and intermediate precision of retention time, peak width, and peak asymmetry measured for NCHs in spiked surface water Flowrate 300 μL/min, 5 μL injection volume. Separation column: Primesep 200, Gradient according to ESM Table S3. MS condition according to ESM Table S4

Compound	Retention time		Peak width		Peak asymmetry	
	Repeatability (%RSD)	Intermediate precision (%RSD)	Repeatability (%RSD)	Intermediate precision (%RSD)	Repeatability (%RSD)	Intermediate precision (%RSD)
Imidazole	0.15	0.4	3.89	3.85	0.04	0.05
Pyrazole	0.15	0.28	4.45	5.07	0.04	0.04
Pyridine	0.08	0.18	2.40	2.44	0.02	0.02
Pyridazine	23.6	24.9	23.37	30.74	0.37	0.51
Piperidine	0.09	0.2	2.14	2.15	0.03	0.03

### Application in ozonation of NCH-containing matrix

**Fig. 3:**
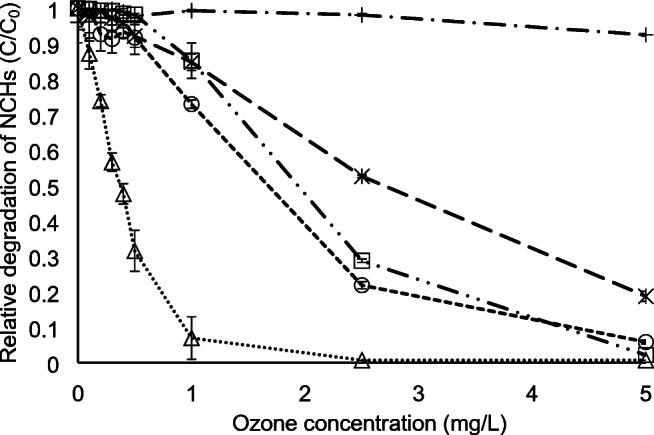
Relative degradation of NCHs with ozone in a surface water matrix spiked with a mixture of compounds with a concentration of 100 μg/L of each measured by LC-MS method. Imidazole (triangles, dotted line), pyrazole (circles, dashed line), pyridine (stars, long dashed line), pyridazine (crosses, long dash-dotted line), and piperidine (squares, long dash double dotted line). Error bars are the standard deviation of triplicate samples. Flowrate 300 μL/min, 5 μL injection volume. Separation column: Primesep 200, Gradient according to ESM Table S3. MS condition according to ESM Table S4. Note that recovery of pyridazine was 30%

As mentioned before, NCHs have to be abated during wastewater treatment and in drinking water since NCHs may contaminate drinking water resources and ozonation is one of the methods [11, 18]. A set of experiments was designed to follow the reduction of NCHs using ozone, in which river water was spiked to 100 μg/L NCHs each. Afterwards, the solution was divided into 10 mL samples in which ozone was dosed. The samples were analyzed with the developed LC-MS method at minimum 24 h after ozone was dosed for assuring full ozone depletion (note that typical lifetimes of ozone in ozonation are below 1 h). Figure 3 shows that ozonation has different efficiencies in the reduction of NCHs. Imidazole is the most efficiently eliminated compound, followed by pyrazole. Both compounds may be degraded at typical conditions of drinking water ozonation. The other compounds seem to be much more ozone refractory. Pyrazole, piperidine, and pyridine require elevated ozone dosages, which could result in undesired by-product formation. Pyridazine was not degraded in the present experiment, which covers a wide range of ozone dosages applied in drinking water. The results show that the developed method can be readily applied to analyze NCHs in real water samples and monitor their degradation in oxidation processes.

## Conclusion

The present work has shown that the MMLC is a complementary alternative for HILIC in the separation of polar compounds such as NCHs. Decent knowledge on the separation mechanisms largely helps to develop and optimize methods for their separation. The successful measurement of the selected NCHs should also facilitate the use of MMLC for the quantification of other NCHs and polar compounds that are difficult to retain in RPLC, such as 1,2,4-Triazole. Moreover, short run time, no sample preparation, and internal standard make this method, time-efficient, robust, and cheap. However, to further increase sensitivity for environmental monitoring, higher injection volumes or enrichment methods that were recently reported such as multi-layer solid-phase extraction [32], vacuum-assisted evaporative [61], or freeze-drying [62] might be necessary. It was demonstrated that the present method could be used to determine NCHs in trace concentrations in real water samples and to monitor their abatement during water treatment processes such as oxidation processes.

## Electronic supplementary material

ESM 1 (PDF 526 kb)

## References

[1] Grosser RJ, Vestal JR, Warshawsky D. Mineralization of polycyclic and N-heterocyclic aromatic compounds in hydrocarbon-contaminated soils. Environ Toxicol Chem. 1995;14(3):375–82.

[2] Ansari A, Ali A, Asif M (2016). Shamsuzzaman. Review: biologically active pyrazole derivatives. New J Chem.

[3] Berger U, Ost N, Sättler D, Schliebner I, Kühne R, Schüürman G, et al. Assessment of persistence, mobility and toxicity (PMT) of 167 REACH registered substances. Umweltbundesamt (UBA). 9/2018.

[4] Sims GK, O’Loughlin EJ, Crawford RL. Degradation of pyridines in the environment. Crit Rev Environ Control. 1989;19(4):309–40.

[5] Pagga U, Bachner J, Strotmann U (2006). Inhibition of nitrification in laboratory tests and model wastewater treatment plants. Chemosphere..

[6] Bi E, Schmidt TC, Haderlein SB (2006). Sorption of heterocyclic organic compounds to reference soils: column studies for process identification. Environ Sci Technol.

[7] Broholm MM, Broholm K, Arvin E. Sorption of heterocyclic compounds on natural clayey till. J Contam Hydrol. 1999;39(3–4):183–200.

[8] Lee ST, Rhee SK, Lee GM (1994). Biodegradation of pyridine by freely suspended and immobilized Pimelobacter sp. Appl Microbiol Biotechnol.

[9] Roper WL. Toxicological profile for pyridine. Agency for Toxic Substances and Disease Registry, US Public Health Service. 1992. 37797098

[10] Zamfirescu D, Grathwohl P. Occurrence and attenuation of specific organic compounds in the groundwater plume at a former gasworks site. J Contam Hydrol. 2001;53(3–4):407–27. 10.1016/s0169-7722(01)00176-0 11820480

[11] Fleig M, Scheurer M, Schmidt CK. Wesentliche Ergebnisse aus dem ARW-Untersuchungs - programm 2018. In: Jahresbericht. Köln: Arbeitsgemeinschaft Niederrhein-Wasserwerke; 2018. p. 15–54.

[12] Stroomberg GJ. RIWA position INEOS permit application ozone installation. 2017. Available from: https://www.riwa-rijn.org/wp-content/uploads/2018/02/RIWA-position-INEOS-permit-application-ozone-installation.pdf. Accessed on 6 Nov 2019.

[13] Stuber HA, Leenheer JA (1983). Selective concentration of aromatic bases from water with a resin adsorbent. Anal Chem.

[14] Lamouroux C, Foglia G, Le Rouzo G. How to separate ionic liquids: use of hydrophilic interaction liquid chromatography and mixed mode phases. J Chromatogr A. 2011;1218(20):3022–8. 10.1016/j.chroma.2011.03.053 21497357

[15] Padoley KV, Mudliar SN, Pandey RA. Heterocyclic nitrogenous pollutants in the environment and their treatment options – an overview. Bioresour Technol. 2008;99(10):4029–43. 10.1016/j.biortech.2007.01.047 17418565

[16] Soltanzadeh Z, Imanzadeh G, Noroozi-Pesyan N, Şahin E (2017). Green synthesis of pyrazole systems under solvent-free conditions. Green Chem Lett Rev.

[17] Vitaku E, Smith DT, Njardarson JT. Analysis of the Structural Diversity, Substitution Patterns, and Frequency of Nitrogen Heterocycles among U.S. FDA Approved Pharmaceuticals. J Med Chem. 2014;57(24):10257–74. 10.1021/jm501100b 25255204

[18] Lowenbach W, Schlesinger J, King J. Toxic pollutant Identificatio: acrylonitrile manufacturing. McLean, Virginia: EPA IMPQE Series; 1978.

[19] Wang Z, Xu X, Yang F, Tan Z, Chen J (2015). Biodegradability of some nitrogenous heterocyclic compounds and co-degradationwith phenol by denitrifiers in anoxic sludge reactor. Water Sci Technol.

[20] Padoley KV, Rajvaidya AS, Subbarao TV, Pandey RA (2006). Biodegradation of pyridine in a completely mixed activated sludge process. Bioresour Technol.

[21] Lide DR. CRC Handbook of chemistry and physics. 90th ed. Boca Raton: CRC Press; 2010.

[22] Umweltbundesamt. Bundesanzeiger. 2017 Aug;August:1–156. Available from: https://www.bundesanzeiger.de/ebanzwww/wexsservlet?session.sessionid=6a5a4d191d0f9dba22ec6b7ee3ec70cf&page.navid=official_view_publicationtoofficial_start&fts_search_list.destHistoryId=30586. Accessed 29 Sep 2019.

[23] Neumann M, Schliebner I. Protecting the sources of our drinking water. second, revised edition. German Environment Agency; 2017. Available from: https://www.umweltbundesamt.de/en/publikationen/protecting-the-sources-of-our-drinking-water-from Accessed 21 Oct 2019.

[24] Umweltbundesamt. Liste der nach GOW bewerteten Stoffe. 2019. p. 1–2. Available from: https://www.umweltbundesamt.de/dokument/liste-nach-gow-bewerteten-stoffe. Accessed Oct 22 2019.

[25] European Chemicals Agency. Information from Registration Dossiers. 2019. Available from: https://echa.europa.eu//-/registered-dossier/23403/5/3/1. Accessed 22 Oct 2019.

[26] Tekle-Röttering A, Jewell KS, Reisz E, Lutze HV, Ternes TA, Schmidt W, et al. Ozonation of piperidine, piperazine and morpholine: kinetics, stoichiometry, product formation and mechanistic considerations. Water Res. 2016;88:960–71. 10.1016/j.watres.2015.11.027 26624229

[27] Tekle-Röttering A, Reisz E, Jewell KS, Lutze HV, Ternes TA, Schmidt W, et al. Ozonation of pyridine and other N-heterocyclic aromatic compounds: kinetics, stoichiometry, identification of products and elucidation of pathways. Water Res. 2016;102:582–93. 10.1016/j.watres.2016.06.021 27448509

[28] McCalley DV (2010). The challenges of the analysis of basic compounds by high performance liquid chromatography: some possible approaches for improved separations. J Chromatogr A.

[29] Lobrutto R. Retention of ionizable compounds in HPLC. Seton Hall University Dissertations and Theses (ETDs); 2000.

[30] Zhu Y, Ren H, Wei Y, Bie Z, Ji L. Determination of imidazole, 4-methylimidazole, and 2-methylimidazole in cigarette additives by ultra-high performance liquid chromatography. Anal Lett. 2015;48(17):2708–14.

[31] Reemtsma T, Berger U, Arp HPH, Gallard H, Knepper TP, Neumann M, et al. Mind the gap: persistent and mobile organic compounds—water contaminants that slip through. Environ Sci Technol. 2016;50(19):10308–15. 10.1021/acs.est.6b03338 27571393

[32] Köke N, Zahn D, Knepper TP, Frömel T. Multi-layer solid-phase extraction and evaporation—enrichment methods for polar organic chemicals from aqueous matrices. Anal Bioanal Chem. 2018;410(9):2403–11. 10.1007/s00216-018-0921-1 29435633

[33] Scheurer M, Brauch H-J, Schmidt CK, Sacher F. Occurrence and fate of nitrification and urease inhibitors in the aquatic environment. Environ Sci Process Impacts. 2016;18(8):999–1010. 10.1039/c6em00014b 27058057

[34] Schmidt TC (2018). Recent trends in water analysis triggering future monitoring of organic micropollutants. Anal Bioanal Chem.

[35] Stepnowski P, Mrozik W (2005). Analysis of selected ionic liquid cations by ion exchange chromatography and reversed-phase hihg performance liquid chromatography. J Sep Sci.

[36] Hawkins CA, Rud A, Guthrie ML, Dietz ML. Rapid quantification of imidazolium-based ionic liquids by hydrophilic interaction liquid chromatography: methodology and an investigation of the retention mechanisms. J Chromatogr A. 2015;1400:54–64. 10.1016/j.chroma.2015.04.047 25979537

[37] Zhang K, Liu X. Reprint of “mixed-mode chromatography in pharmaceutical and biopharmaceutical applications.”. J Pharm Biomed Anal. 2016;130:19–34. 10.1016/j.jpba.2016.09.013 27645457

[38] Montes R, Aguirre J, Vidal X, Rodil R, Cela R, Quintana JB (2017). Screening for polar chemicals in water by trifunctional mixed-mode liquid chromatography-high resolution mass spectrometry. Environ Sci Technol.

[39] Li J, Shao S, Jaworsky MS, Kurtulik PT (2008). Simultaneous determination of cations, zwitterions and neutral compounds using mixed-mode reversed-phase and cation-exchange high-performance liquid chromatography. J Chromatogr A.

[40] Hoigné J, Bader H (1980). Bestimmung von Ozon und Chlordioxid in Wasser mit der Indigo-Methode. Vom Wasser.

[41] von Sonntag C, von Gunten U. Chemistry of ozone in water and wastewater treatment: IWA publishing; 2012.

[42] Ordoñez EY, Quintana JB, Rodil R, Cela R. Computer assisted optimization of liquid chromatographic separations of small molecules using mixed-mode stationary phases. J Chromatogr A. 2012;1238:91–104. 10.1016/j.chroma.2012.03.055 22494641

[43] Ståhlberg J. Retention models for ions in chromatography. J Chromatogr A. 1999;855(1):3–55. 10.1016/s0021-9673(99)00176-4 10514972

[44] Subirats X, Bosch E, Rosés M (2009). Retention of ionisable compounds on high-performance liquid chromatography XVIII: pH variation in mobile phases containing formic acid, piperazine, tris, boric acid or carbonate as buffering systems and acetonitrile as organic modifier. J Chromatogr A.

[45] DIN. 38402–51 Deutsche Einheitsverfahren zur Wasser-, Abwasser und Schlammuntersuchung – Allgemeine Angaben (Gruppe A) – Teil 51: Kalibrierung von Analysenverfahren – Lineare Kalibrierfunktion (A 51). 2014.

[46] Andrade JM, Gómez-Carracedo MP (2013). Notes on the use of Mandel’s test to check for nonlinearity in laboratory calibrations. Anal Methods.

[47] EPA US. Definition and procedure for the determination of the method detection limit, Revision 2. Environmental Protection Agency EPA. 2016.

[48] Eurachem. Eurachem guide: the fitness for purpose of analytical methods – a laboratory guide to method validation and related topics. 2014;1–62.

[49] Buszewski B, Noga S (2012). Hydrophilic interaction liquid chromatography (HILIC)-a powerful separation technique. Anal Bioanal Chem.

[50] Balkatzopoulou P, Fasoula S, Gika H, Nikitas P, Pappa-Louisi A. Retention prediction of highly polar ionizable solutes under gradient conditions on a mixed-mode reversed-phase and weak anion-exchange stationary phase. J Chromatogr A. 2015;1396:72–6. 10.1016/j.chroma.2015.03.082 25900744

[51] Kloepfer A, Quintana JB, Reemtsma T. Operational options to reduce matrix effects in liquid chromatography–electrospray ionisation-mass spectrometry analysis of aqueous environmental samples. J Chromatogr A. 2005;1067(1–2):153–60. 10.1016/j.chroma.2004.11.101 15844520

[52] EPA US. Subchapter D — Water programs ( continued ) PART 136—guidelines establishing test procedures for the analysis of pollutants. Water Pollut Control. 2003. Available from: https://www.ecfr.gov/cgi-bin/text-idx?SID=a6bb8a02b6d783f9356758b5ff0ed106&mc=true&node=pt40.25.136&rgn=div5#se40.25.136_15. Accessed 22 Oct 2019.

[53] Yamini Y, Seidi S, Pourali A, Rezazadeh M (2015). Electrical field-stimulated liquid-phase microextraction for trace analysis of pyridine and its derivatives in cigarette extract. J Iran Chem Soc.

[54] Orazbayeva D, Kenessov B, Psillakis E, Nassyrova D, Bektassov M. Determination of transformation products of unsymmetrical dimethylhydrazine in water using vacuum-assisted headspace solid-phase microextraction. J Chromatogr A. 2018;1555:30–6. 10.1016/j.chroma.2018.04.048 29716735

[55] LANUV. ECHO-Stoffbericht Pyrazol. p. 1–13. Available from: https://www.lanuv.nrw.de/fileadmin/lanuv/analytik/pdf/ECHO_Pyrazol_2017a.pdf. Accessed 29 Sept 2019.

[56] Zhang XX, Liu YQ, Yu H, Zhang RQ. Rapid and simultaneous determination of piperidinium and pyrrolidinium ionic liquid cations by ion pair chromatography coupled with direct conductivity detection. Chin Chem Lett. 2017;28(1):126–30.

[57] Huang X, Yu H, Dong YJ. Rapid and simultaneous determination of imidazolium and pyridinium ionic liquid cations by ion-pair chromatography using a monolithic column. Chin Chem Lett. 2012;23(7):843–6.

[58] Kruve A, Leito I (2013). Comparison of different methods aiming to account for/overcome matrix effects in LC/ESI/MS on the example of pesticide analyses. Anal Methods.

[59] Baken K, Kolkman A, van Diepenbeek P, Ketelaars H, van Wezel A. Signalering van “overige antropogene stoffen”, en dan? De pyrazool- casus. 2016. Available from: https://www.h2owaternetwerk.nl/images/H2O-Online_1609-03_Pyrazool_II_-_Bakenetal.pdf. Accessed 07 Nov 2019.

[60] Trufelli H, Palma P, Famiglini G, Cappiello A. An overview of matrix effects in liquid chromatography-mass spectrometry. Mass Spectrom Rev. 2011;30(3):491–509. 10.1002/mas.20298 21500246

[61] Mechelke J, Longrée P, Singer H, Hollender J. Vacuum-assisted evaporative concentration combined with LC-HRMS/MS for ultra-trace-level screening of organic micropollutants in environmental water samples. Anal Bioanal Chem. 2019;411(12):2555–67. 10.1007/s00216-019-01696-3 PMC6470124 30854597

[62] Montes R, Rodil R, Cela R, Quintana JB (2019). Determination of persistent and mobile organic contaminants (PMOCs) in water by mixed-mode liquid chromatography-tandem mass spectrometry. Anal Chem.

