# Supplementary material for: Development of an LC-MS method for determination of nitrogen-containing heterocycles using mixed-mode liquid chromatography
Source: Anal Bioanal Chem. 2020 May 26;412(20):4921–30. doi: 10.1007/s00216-020-02665-x (PMC7334287; doi:10.1007/s00216-020-02665-x)
Supplement: Supplementary file 1 — (PDF 526 kb) [file 216_2020_2665_MOESM1_ESM.pdf]

Analytical and Bioanalytical Chemistry

Electronic Supplementary Material

Development of an LC-MS method for determination of nitrogen-containing heterocycles using mixed-mode liquid chromatography

Mohammad Sajjad Abdighahroudi, Holger V. Lutze, Torsten C. Schmidt

Table S1 Chromatographic parameters used for investigation of the separation performance

Parameter	Symbol used	Unit	Measurement
Void time	t_0	min	By injecting NaBr
Retention	t_R	min	
Retention factor	k'	-	$k' = \frac{t_R - t_0}{t_0}$
Peak width	W_{50}	min	At 50% height
Peak asymmetry	A_s	-	At 10% height
Selectivity	α	-	Ratio of the retention factor to the retention factor of the previous peak
Efficiency	N	Plates/Column	$5{,}54 \times (\frac{t_R}{W_{50}})^2$
Resolution	R_s	-	$\frac{1}{4} \times \sqrt{N} \times \frac{k'}{(k'+1)} \times \frac{(\alpha - 1)}{\alpha}$

Table S2 Empirical constants calculated to describe retention factor (log(k')) in isocratic elution of NCHs at different ACN volume fractions according to Equation 4 in the main text.. Eluent A: Water + 0.1% FA, Eluent B: ACN Flowrate 300 µL/min, 5 µL injection volume. Separation column: Primesep 200

Compound	m_1	m_2	C	SSE	R^2
Imidazole	2.63 ± 0.37	4.31 ± 0.45	-1.83 ± 0.34	0.00997	0.979
Pyrazole	2.48 ± 0.24	1.75 ± 0.31	-1.85 ± 0.23	0.00457	0.991
Pyridine	2.76 ± 0.66	4.16 ± 0.81	-1.72 ± 0.6	0.03187	0.901
Pyridazine	3 ± 0.41	2.81 ± 0.51	-2.44 ± 0.38	0.01254	0.962
Piperidine	3.18 ± 0.61	4.76 ± 0.75	2.09 ± 0.56	0.02788	0.932

Table S3 Gradient of the separation of NCHs in method optimized for surface water measurement

Time	Eluent A Water + 0.1% FA [%]	Eluent B ACN + 0.2% FA [%]
0	95	5
5	95	5
8	50	50
15	50	50
15.01	95	5
25	95	5

Table S4 MS settings for measurement of NCHs

	**Signal 1 TIC**	**Signal 2 SIM**		**Signal 3 SIM**	
Cycle percent	**20**	**40**		**40**	
Mass range	65-90	81	80	69	86.10
Time span	0-25	0-8	8-25	0-10.5	10.5-25

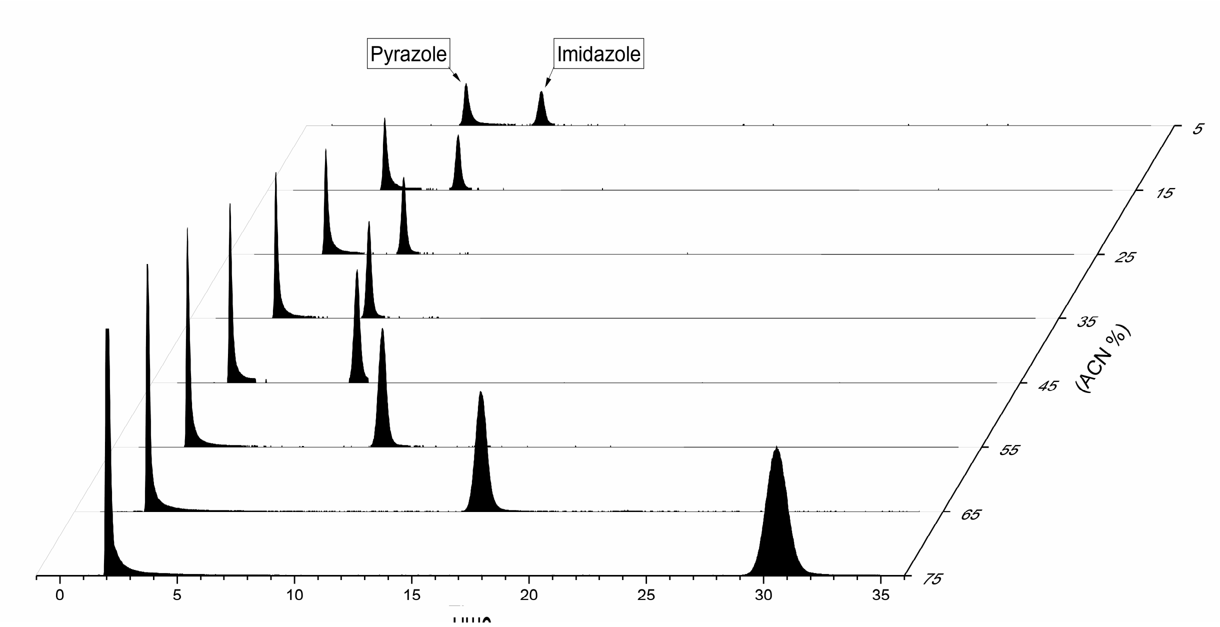

Fig. S1 EIC (m/z = 69), of pyrazole and imidazole at different δ_{ACN} (isocratic) showing different modes of interaction. NHCs concentration 1000 µg/L, Eluent A: Water + 0.1 FA, Eluent B: ACN Flowrate 300 µL/min, 5 µL injection volume. Primesep 200

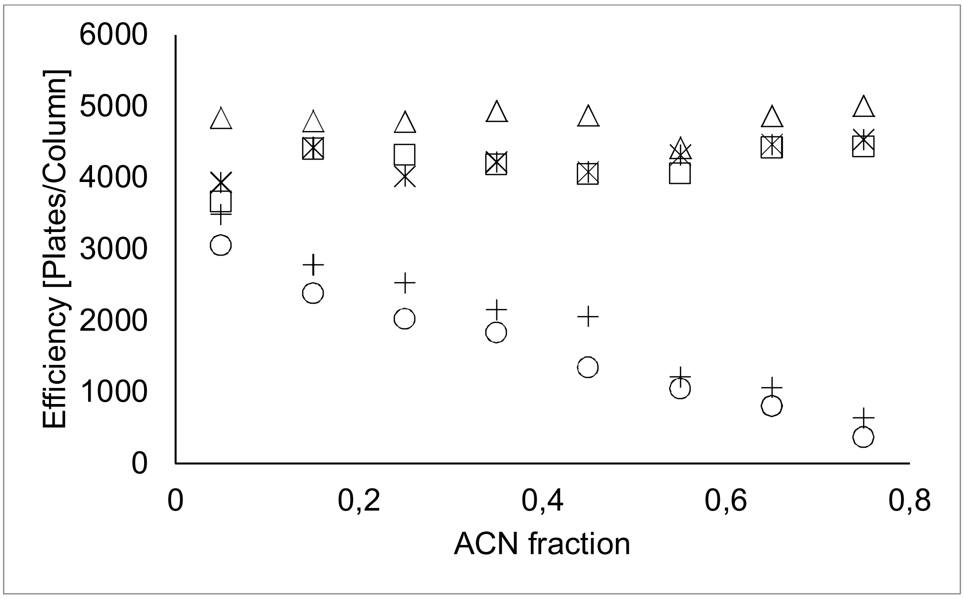

Fig. S2 Efficiency in isocratic elution of NCHs at different ACN volume fractions. Imidazole (triangles) pyrazole (circles) pyridine (stars) pyridazine (crosses), and piperidine (squares) Eluent A: Water + 0.1% FA, Eluent B: ACN Flowrate 300 µL/min, 5 µL injection volume. Separation column: Primesep 200

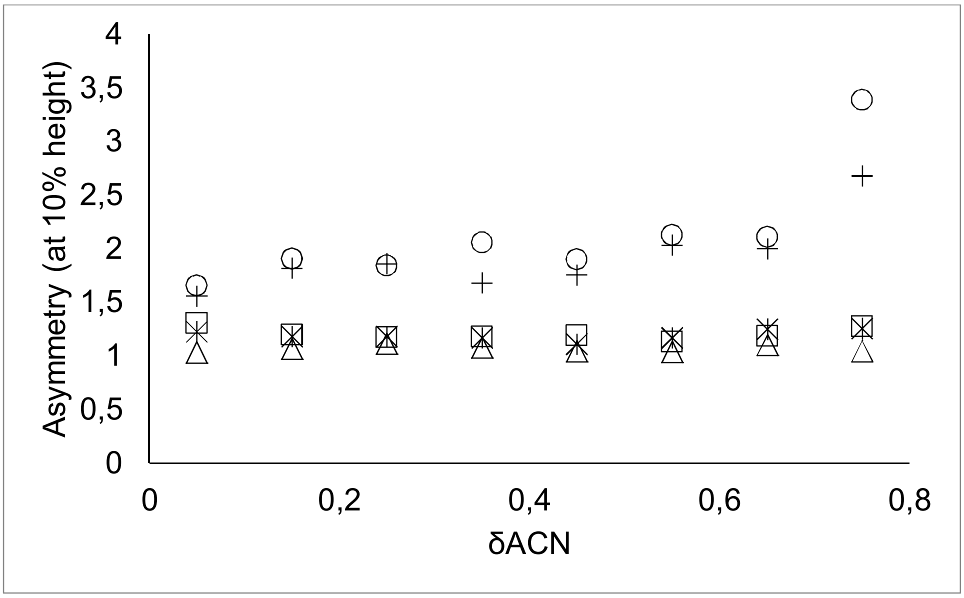

Fig. S3 Peak asymmetry in isocratic elution of NHCs at different ACN volume fractions. Imidazole (triangles) Pyrazole (circles) Pyridine (stars) Pyridazine (crosses) and piperidine (squares) Eluent A: Water + 0.1 Formic acid, Eluent B: ACN Flowrate 300 µL/min, 5 µL injection volume. Separation column: Primesep 200

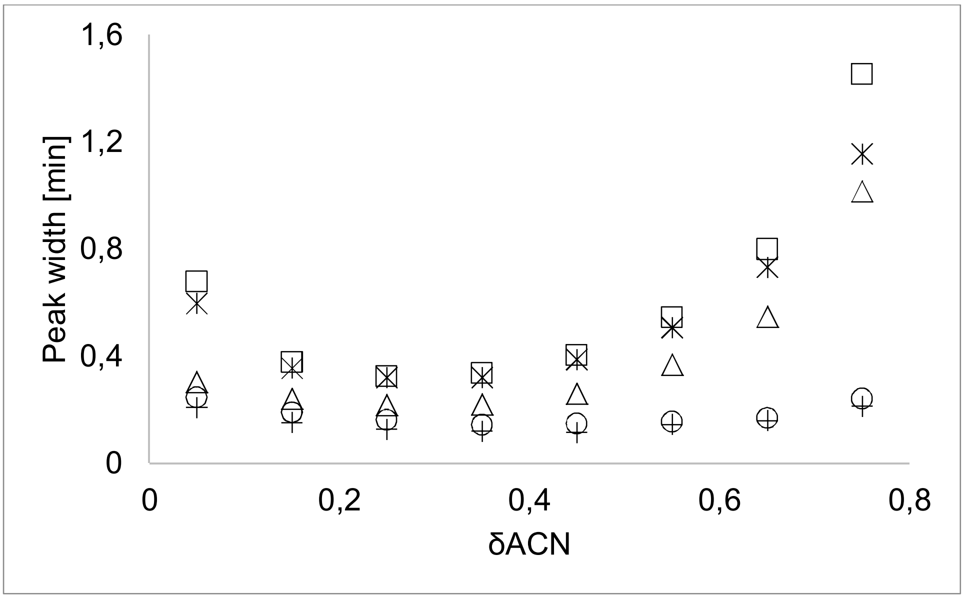

Fig. S4 Peak width in isocratic elution of NHCs at different ACN volume fractions. Imidazole (triangles) Pyrazole (circles) Pyridine (stars) Pyridazine (crosses) and piperidine (squares) Eluent A: Water + 0.1 Formic acid, Eluent B: ACN Flowrate 300 µL/min, 5 µL injection volume. Separation column: Primesep 200

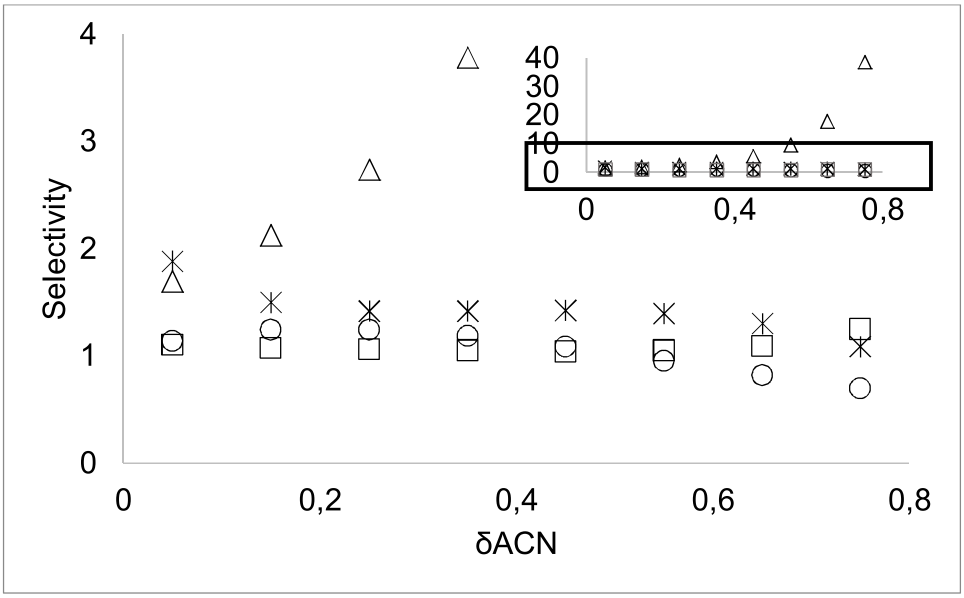

Fig. S5 Selectivity in isocratic elution of NHCs at different ACN volume fractions. (main figure represents an enlarged view of the black-bordered box of the insert) Imidazole (triangles) Pyrazole (circles) Pyridine (stars) Pyridazine (crosses) and piperidine (squares) Eluent A: Water + 0.1 Formic acid, Eluent B: ACN Flowrate 300 µL/min, 5 µL injection volume. Separation column: Primesep 200

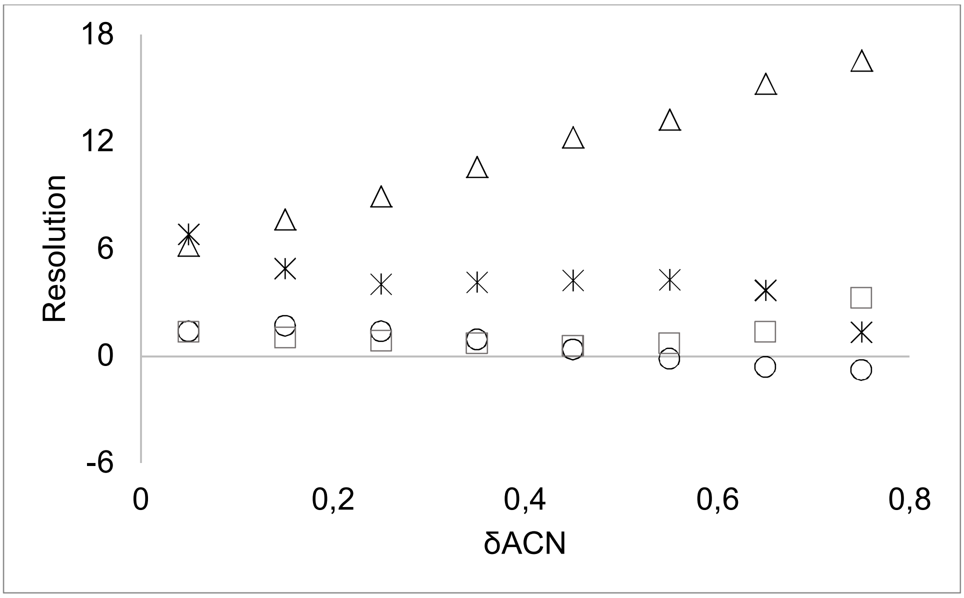

Fig. S6 Resolution in isocratic elution of NHCs at different ACN volume fractions. Imidazole (triangles) Pyrazole (circles) Pyridine (stars) Pyridazine (crosses) and piperidine (squares) Eluent A: Water + 0.1 Formic acid, Eluent B: ACN Flowrate 300 µL/min, 5 µL injection volume. Separation column: Primesep 200

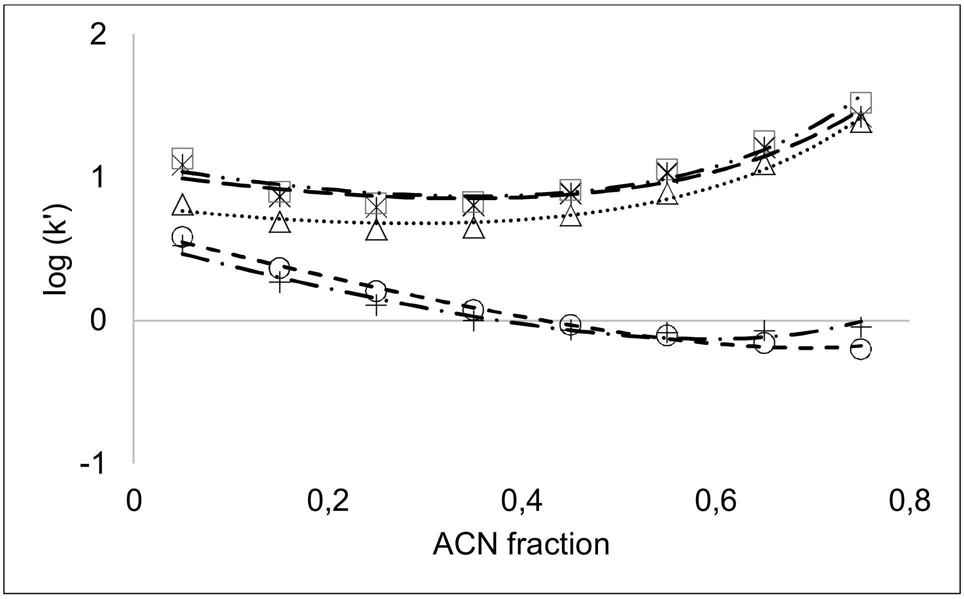

Fig. S7 Experimental and calculated logarithm of retention factor (log(k')) in isocratic elution of NCHs at different ACN volume fractions. Imidazole (triangles, dotted line) pyrazole (circles, dashed line) pyridine (stars, long dashed line) pyridazine (crosses, long dash-dotted line), and piperidine (squares, long dash double dotted line). Eluent A: Water + 0.1% FA, Eluent B: ACN Flowrate 300 µL/min, 5 µL injection volume. Separation column: Primesep 200

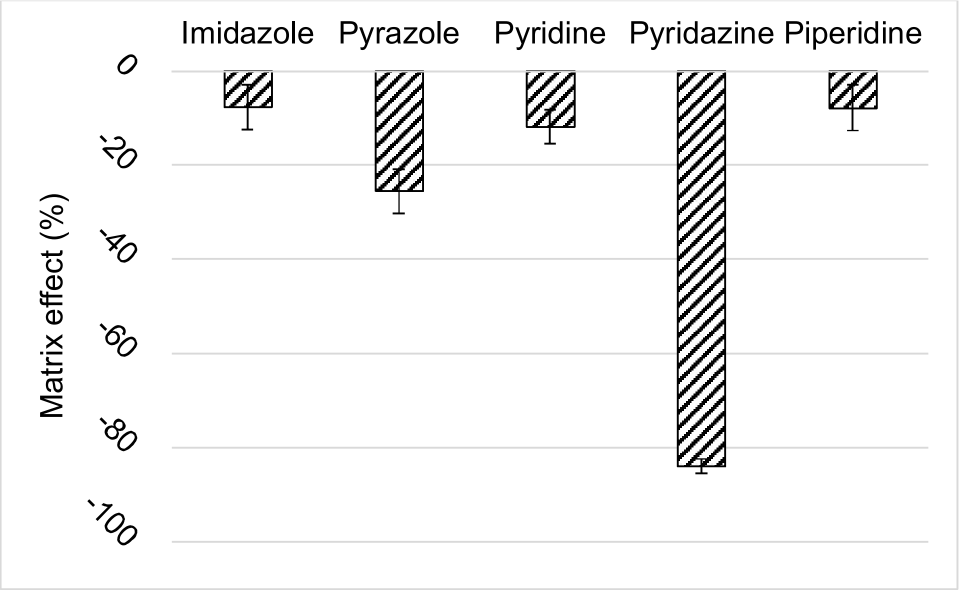

Fig. S8 Matrix effect observed in the measurement of NCHs in spiked surface water showing ion suppression for all NCHs. Flowrate 300 µL/min, 5 µL injection volume. Separation column: Primesep 200, Gradient according to Table S2. MS condition according to Table S3
